# Validation of SARS-CoV-2 pooled testing for surveillance using the Panther Fusion^®^ system: Impact of pool size, automation, and assay chemistry

**DOI:** 10.1371/journal.pone.0276729

**Published:** 2022-11-07

**Authors:** Rudolph Park, Prabha Chandrasekaran, Heather Hernandez, Ines Lakhal-Naouar, Kristina K. Peachman, Holly R. Hack, Dante Coleman, Jason Ouellette, Janice M. Darden, Oussama M’hamdi, Victor A. Sugiharto, Hua-Wei Chen, Megan A. Schilling, Mark P. Simons, Natalie D. Collins, Yuliya S. Johnson, Linda L. Jagodzinski, Sheila A. Peel

**Affiliations:** 1 Diagnostics and Countermeasures Branch (DCB), Walter Reed Army Institute of Research, Silver Spring, MD, United States of America; 2 Henry M. Jackson Foundation for the Advancement of Military Medicine, Bethesda, MD, United States of America; 3 Naval Infectious Diseases Diagnostic Laboratory, Naval Medical Research Center, Silver Spring, MD, United States of America; 4 Viral Disease Branch (VDB), Walter Reed Army Institute of Research, Silver Spring, MD, United States of America; 5 Operationally Relevant Infections Department, Naval Medical Research Center, Silver Spring, MD, United States of America; Waseda University: Waseda Daigaku, JAPAN

## Abstract

Combining diagnostic specimens into pools has been considered as a strategy to augment throughput, decrease turnaround time, and leverage resources. This study utilized a multi-parametric approach to assess optimum pool size, impact of automation, and effect of nucleic acid amplification chemistries on the detection of SARS-CoV-2 RNA in pooled samples for surveillance testing on the Hologic Panther Fusion^®^ System. Dorfman pooled testing was conducted with previously tested SARS-CoV-2 nasopharyngeal samples using Hologic’s Aptima^®^ and Panther Fusion^®^ SARS-CoV-2 Emergency Use Authorization assays. A manual workflow was used to generate pool sizes of 5:1 (five samples: one positive, four negative) and 10:1. An automated workflow was used to generate pool sizes of 3:1, 4:1, 5:1, 8:1 and 10:1. The impact of pool size, pooling method, and assay chemistry on sensitivity, specificity, and lower limit of detection (LLOD) was evaluated. Both the Hologic Aptima^®^ and Panther Fusion^®^ SARS-CoV-2 assays demonstrated >85% positive percent agreement between neat testing and pool sizes ≤5:1, satisfying FDA recommendation. Discordant results between neat and pooled testing were more frequent for positive samples with C_T_>35. Fusion^®^ C_T_ (cycle threshold) values for pooled samples increased as expected for pool sizes of 5:1 (C_T_ increase of 1.92–2.41) and 10:1 (C_T_ increase of 3.03–3.29). The Fusion^®^ assay demonstrated lower LLOD than the Aptima^®^ assay for pooled testing (956 vs 1503 cp/mL, pool size of 5:1). Lowering the cut-off threshold of the Aptima^®^ assay from 560 kRLU (manufacturer’s setting) to 350 kRLU improved the assay sensitivity to that of the Fusion^®^ assay for pooled testing. Both Hologic’s SARS-CoV-2 assays met the FDA recommended guidelines for percent positive agreement (>85%) for pool sizes ≤5:1. Automated pooling increased test throughput and enabled automated sample tracking while requiring less labor. The Fusion^®^ SARS-CoV-2 assay, which demonstrated a lower LLOD, may be more appropriate for surveillance testing.

## Introduction

The dire need for early detection and surveillance of SARS-CoV-2 infections led laboratories and global diagnostic manufacturers to rapidly develop approaches for virus detection [1, 2]. The Food and Drug Administration (FDA) has subsequently permitted the use of over 269 molecular assays under its Emergency Use Authorization (EUA) mechanism [3]. In order to contain the SARS-CoV-2 pandemic, surveillance testing has been implemented so that asymptomatic individuals can be identified, treated, and isolated to limit further spread [4, 5]. Asymptomatic spread is uniquely relevant to SARS-CoV-2, as most other disease infections are symptomatic with the onset of viremia [6].

One strategy to expand surveillance testing is to implement pooled sample testing, which involves combining multiple samples into a single test to reduce the turnaround time, cost, and laboratory resources required [7–9]. The performance of pooled testing depends on multiple parameters including disease prevalence, pool size, pooling method, and assay chemistry [10]. Pool sizes up to 64 [11–13] have been recommended for SARS-CoV-2 surveillance testing in low disease prevalence conditions. Automated pooled sample generation can further increase surveillance testing throughput and can reduce pipetting errors and cross contamination, but it requires investment in facility preparation, liquid handling capability, and software development and verification.

In addition to pool size and pooling method, assay chemistry can influence pooled testing performance. Currently, real-time reverse transcription-polymerase chain reaction (RT-PCR) assays are the most common type of molecular test for SARS-CoV-2 diagnosis. However, transcription mediated amplification (TMA) is an alternative nucleic acid amplification chemistry that offers some advantages over RT-PCR. TMA utilizes RNA polymerase to generate many RNA transcripts from a DNA template at a fixed temperature, unlike RT-PCR which uses a temperature sensitive DNA polymerase to generate a single copy of a DNA template per thermal cycle.

This study was designed to evaluate automated pooled testing for surveillance of SARS-CoV-2 infections in the Military Health System. We compared a range of pool sizes using manual and automated methods for pooling samples followed by both RT-PCR and TMA assay testing. Specifically, we evaluated pooled sample testing with both chemistries using the Hologic, Inc. (Marlborough, MA, USA) Panther Fusion^®^ SARS-CoV-2 assay (real-time RT-PCR) and the Hologic Aptima^®^ SARS-CoV-2 assay (TMA). Both molecular assays are approved for use under EUA by the FDA and were run on the same Panther Fusion^®^ system.

## Materials and methods

### Clinical discard samples

For the manual pooling study, 1652 post residual clinical test samples were received under a minimal risk Human Subjects Research study approved by the Walter Reed Army Institute of Research (WRAIR) Institutional Review Board (WRAIR #2810). Nasopharyngeal (NP) swabs were collected in Viral Transport Media (VTM). Due to supply chain disruptions multiple VTM sources were used but all were cleared by the FDA. Diagnostic testing was performed at the Naval Infectious Diseases Diagnostics Laboratory (NIDDL) within the Naval Medical Research Center (NMRC), using CDC 2019-Novel Coronavirus Real-Time RT-PCR Diagnostic Panel or ThermoFisher TaqPath™ COVID-19 Combo Kit assays. Post residual test samples were stored at -80°C, then transferred to the Department of Research, Assessment and Development, Diagnostics and Countermeasures Branch, Center for Infectious Diseases, Walter Reed Army Institute of Research. In total, 106 SARS-CoV-2 positive and 308 negative post residual samples were used for testing. To evaluate assay specificity, 32 negative samples (28 from NIDDL and 4 purchased from BioChemed (Winchester, VA)) were also subjected to pooled testing. For the automated pooling study, 2028 de-identified post residual test samples were acquired from Walter Reed National Military Medical Center (WRNMMC). The EUA Roche cobas^®^ SARS-CoV-2 test was used as the reference clinical diagnostic assay. A total of 107 positive samples and 450 negative samples were used for generating pools; details of the samples are listed in S1 Table. Both automated and manual pooled testing schemas used samples with C_T_ ranging from 15 to >35, with ~15% low viral load samples (C_T_>35). The samples used to support automated pooling came from a cohort with a higher proportion (56%) of moderate viral load samples (25<C_T_<35) than the cohort that supported the manual pooling study (35% moderate viral load).

### Manual sample pooling

Previously identified positive SARS-CoV-2 post residual clinical test samples were tested neat and in pool sizes of 5:1 and 10:1. For neat testing, 500 μL of each sample was pipetted directly into a Panther Fusion^®^ Specimen Lysis Tube (SLT) containing 0.71 mL of Specimen Transport Medium (STM). For pooled sample testing, a two-step pooling process was performed in which the negative samples were first pooled into a sterile, RNase/DNase-free tube, then pipetted into an SLT. Pools were completed by pipetting a positive sample into the SLT. All samples were pipetted in equal volume (100 μL for pools of 5:1, 50 μL for pools of 10:1) to create a final pool volume of 500 μL. A one-step pooling process was performed to determine if false positive results could be reduced by directly pipetting negative samples into SLTs. The schemas for manual pooling are presented in S1 Fig.

### Automated sample pooling

Post residual clinical test samples were tested neat or in pool sizes of 3:1, 4:1, 5:1, 8:1 and 10:1. Neat and pooled samples were prepared using a Tecan (Zürich, Switzerland) Freedom EVO 150 robotic liquid handler equipped with an Air Liquid Handler and PosID3 barcode reader. The EVO 150 was housed inside a Labconco (Kansas City, MO) Logic Vue Class II enclosure. The software (scripts) required to automate pipetting with the Tecan EVO 150 were defined by the authors (DCB), written by Tecan’s Clinical Applications Specialists, and verified by the authors (DCB) and Tecan. The operator enters the pooling parameters (number of samples per pool, number of samples to be prepared, final pool volume), and the script performs the pipetting and sample tracking. Barcoded labels were affixed to the source sample tubes and pooled sample tubes to enable automated sample tracking.

### Hologic Panther^®^ SARS-CoV-2 molecular assays

Two high-throughput, automated SARS-CoV-2 molecular assays were used for this pooling study. The Hologic Panther Fusion^®^ SARS-CoV-2 assay, which utilizes real-time RT-PCR and gives a semi-quantitative result (positive/negative and C_T_), and the Hologic Aptima^®^ SARS-CoV-2 assay, which utilizes TMA [14] and yields a qualitative (positive/negative) result. Results for the Aptima^®^ SARS-CoV-2 assay are expressed as the final amount of amplified target as kilo Relative Light Units (kRLU). The assay results are determined by a cut-off based on the total RLU (>560 kRLU for positive result) and the kinetic curve type. Both assays were performed on the same Panther Fusion^®^ instrument, with neat and pooled samples tested simultaneously. Characteristics for the two Hologic assays are summarized in S2 Table.

### LLOD panel

To evaluate the effect of pooling on the LLOD of both Hologic assays, SARS-CoV-2 samples with varying concentrations of SARS-CoV-2 RNA were generated using heat inactivated SARS-CoV-2 RNA (HK-87, American Type Culture Collection (ATCC), Manassas, VA). Twelve concentrations of RNA from 24,000 copies/mL to 16 copies/mL were prepared in Universal Transport Medium (UTM, brand of VTM trademarked by Copan). Six pool sizes were created using SARS-CoV-2 RNA negative post residual clinical test discards. S3 Table shows the 12 starting concentrations in the “Neat” row. STM/UTM was used to contrive dilution panel concentrations. The 12 starting concentrations (“Neat” row) were contrived by adding a known quantity of the heat inactivated SARS-CoV-2 virus in STM/UTM media. Negative post residual clinical samples were used to generate pools of 3:1, 4:1, 5:1. 8:1 and 10:1 from these 12 starting concentrations.

#### Comparing LLOD with two sources of viral RNA

To quantify the effect of pooling on the LLOD, we contrived a panel with known concentrations of either heat inactivated SARS-CoV-2 virus (HK-87, ATCC, Manassas, VA) or genomic RNA isolated from SARS-CoV-2 (BEI Resources, Manassas, VA).

### False positive testing

To further investigate FPs and prevent bias, negative sample pooled testing was performed using both Aptima^®^ and Fusion^®^ assays. Thirty two (32) samples that had previously tested negative (either at NIDDL or WRNMMC) and also tested negative in our hands with both the Aptima^®^ and Fusion^®^ assays were manually pooled (5:1 and 10:1) using the two-step pooling process. It was hypothesized that pooling directly into the lysis buffer might prevent potential cross reactivity or interference, so we also tested 29 samples (25 negative, 4 positive) with manual pooling (5:1 and 10:1) using both the one-step and two-step pooling processes. Once the automated pooling platform was verified, we performed automated pooling with 40 previously tested negative samples for all pool sizes using the two-step pooling process.

### Statistical analysis

Samples were deemed positive or negative based on the consensus of the original clinical result, the Aptima^®^ SARS-CoV-2 assay result, and the Fusion^®^ SARS-CoV-2 assay result. Confidence intervals (CI) for the likelihood ratios, sensitivity, and specificity calculations, and predictive values are presented as Log_10_, exact Clopper-Pearson and logit CIs, respectively. The reliability for qualitative detection was assessed through Cohen’s kappa coefficient (κ), where values of κ define the following categories: slight (0.0 to 0.2), fair (0.21–0.4), moderate (0.41 to 0.60), substantial (0.61 to 0.8), and almost perfect (0.81–1.0). κ calculations were performed using Python. Passing-Bablok regression analysis was performed by programming in R, version 3.5.1, by using the method comparison regression (mcr) package. The bar graphs and Bland-Altman plots were generated using GraphPad Prism, version 8.4.3. A Freidman test was performed to determine the significance of observed differences in the bar graphs. Statistics on the bar graphs and Bland-Altman plots were performed using GraphPad Prism. CIs were calculated using MedCalc, version 19.4.1. Statistics for Tables 1–4 were calculated using Microsoft Excel. Statistics for Tables 5 and 6 were calculated using Microsoft Excel and MedCalc. All comparisons were two-sided with type 1 error set to 0.05; hence, p-value<0.05 was considered significant.

**Table 1:** PPA between Aptima^®^ SARS-CoV-2 pooled sample test results and neat test results from three SARS-CoV-2 assays.

Manual				Automated						
	Pool Size				Pool Size					
**Assay**	Neat	5:1	10:1	**Assay**	Neat	3:1	4:1	5:1	8:1	10:1
Aptima n = 106	-	89.6	86.8	Aptima n = 95	-	92.6	90.5	89.5	90.5	88.4
CDC, TaqPath, cobas Cut-off = 560 kRLU n = 106	100.0	89.6	86.8	CDC, TaqPath, cobas Cut-off = 560 kRLU n = 107	88.8	82.2	80.4	79.4	80.4	78.5
CDC, TaqPath, cobas Cut-off = 350 kRLU n = 107	100.0	91.6	89.7	CDC, TaqPath, cobas Cut-off = 350 kRLU n = 107	95.3	86.0	86.9	86.9	84.1	85.0

**Table 2:** PPA between Fusion^®^ SARS-CoV-2 pooled test results and neat test results from three SARS-CoV-2 assays.

Manual				Automated						
	Pool Size				Pool Size					
**Assay**	Neat	5:1	10:1	**Assay**	Neat	3:1	4:1	5:1	8:1	10:1
Fusion n = 103	-	96.1	88.3	Fusion n = 105	-	89.5	85.7	85.7	82.9	81.9
CDC, TaqPath, cobas n = 106	97.2	93.4	85.8	CDC, TaqPath, cobas n = 107	98.1	89.7	85.0	85.0	82.2	81.3

**Table 3:** PPA of Aptima^®^ SARS-CoV-2 assay as function of neat Fusion^®^ C_T_.

	Manual		Automated				
**C**_**T**_ **range**	5:1	10:1	3:1	4:1	5:1	8:1	10:1
C_T_<25	100.0%	98.2%	100.0%	100.0%	100.0%	100.0%	100.0%
25<C_T_<35	96.9%	96.9%	100.0%	98.0%	98.0%	98.0%	98.0%
C_T_>35	50.0%	37.5%	22.0%	25.0%	10.0%	25.0%	50.0%

**Table 4:** PPA of Fusion^®^ SARS-CoV-2 assay as function of neat Fusion^®^ C_T_.

	Manual		Automated				
**C**_**T**_ **range**	5:1	10:1	3:1	4:1	5:1	8:1	10:1
C_T_<25	100.0%	98.0%	100.0%	100.0%	100.0%	100.0%	100.0%
25<C_T_<35	93.8%	93.8%	100.0%	100.0%	100.0%	100.0%	96.2%
C_T_>35	87.5%	56.3%	45.0%	25.0%	25.0%	17.7%	20.0%

**Table 5:** Aptima^®^ performance parameters.

Manual			Automated					
	Pool Size			Pool Size				
**Parameter**	5:1	10:1	**Parameter**	3:1	4:1	5:1	8:1	10:1
Sensitivity	0.896	0.868	Sensitivity	0.804	0.832	0.804	0.832	0.785
	.822 to .947	.788 to .926		.716 to .874	.747 to .897	.716 to .874	.747 to .897	.695 to .859
Specificity	0.973	1.000	Specificity	0.980	0.959	0.959	1.000	0.980
	.858 to .999	.905 to 1.00		.891 to .999	.860 to .995	.860 to .995	.927 to 1.00	.891 to .999
PPV	0.990	1.000	PPV	0.989	1.000	0.989	1.000	1.000
	.943 to 1.00	.961 to 1.00		.938 to 1.00	.959 to 1.00	.938 to 1.00	.959 to 1.00	.957 to 1.00
NPV	0.750	0.725	NPV	0.720	0.750	0.716	0.757	0.705
	.597 to .868	.583 to .841		.604 to .818	.634 to .845	.599 to .815	.643 to .849	.591 to .803
kappa κ	0.80	0.77	kappa κ	0.83	0.83	0.80	0.90	0.82
	0.69 to 0.91	0.66 to 0.88		0.73 to 0.93	0.73 to 0.93	0.69 to 0.91	0.81 to 0.98	0.71 to 0.92

Lower number in each section is 95% Confidence Interval value. PPV: Positive Predictive Value, NPV: Negative Predictive Value. The reliability for qualitative detection was assessed through Cohen’s kappa coefficient (κ), where values of κ define the following categories: Slight (0.0 to 0.2), Fair (0.21–0.4), Moderate (0.41 to 0.60), Substantial (0.61 to 0.8), and Almost Perfect (0.81–1.0) agreement.

**Table 6:** Fusion^®^ performance parameters.

Manual			Automated					
	Pool Size			Pool Size				
**Parameter**	5:1	10:1	**Parameter**	3:1	4:1	5:1	8:1	10:1
Sensitivity	0.934	0.858	Sensitivity	0.897	0.850	0.850	0.832	0.813
	.869 to .973	.777 to .919		.824 to .948	.769 to .912	.769 to .912	.747 to .897	.726 to .882
Specificity	0.889	0.925	Specificity	0.959	0.959	0.939	0.959	0.959
	.739 to .969	.796 to .984		.860 to .995	.860 to .995	.831 to .987	.860 to .995	.860 to .995
PPV	0.961	0.989	PPV	1.000	0.989	0.978	0.989	0.989
	.904 to .989	.941 to 1.00		.923 to 1.00	.941 to 1.00	.924 to .997	.940 to 1.00	.938 to 1.00
NPV	0.821	0.712	NPV	0.831	0.768	0.761	0.746	0.730
	.665 to .925	.583 to .841		.717 to .912	.651 to .861	.641 to .857	.629 to .842	.614 to .826
kappa κ	0.84	0.76	kappa κ	0.74	0.68	0.66	0.65	0.61
	0.74 to 0.94	0.64 to 0.87		0.61 to 0.87	0.54 to 0.82	0.51 to 0.80	0.51 to 0.79	0.46 to 0.75

PPV: Positive Predictive Value, NPV: Negative Predictive Value. The reliability for qualitative detection was assessed through Cohen’s kappa coefficient (κ), where values of κ define the following categories: Slight (0.0 to 0.2), Fair (0.21–0.4), Moderate (0.41 to 0.60), Substantial (0.61 to 0.8), and Almost Perfect (0.81–1.0) agreement.

## Results

### Aptima^®^ and Fusion^®^ SARS-CoV-2 RNA detection vs other SARS-CoV-2 EUA assays for neat sample testing

**Fig 1:**
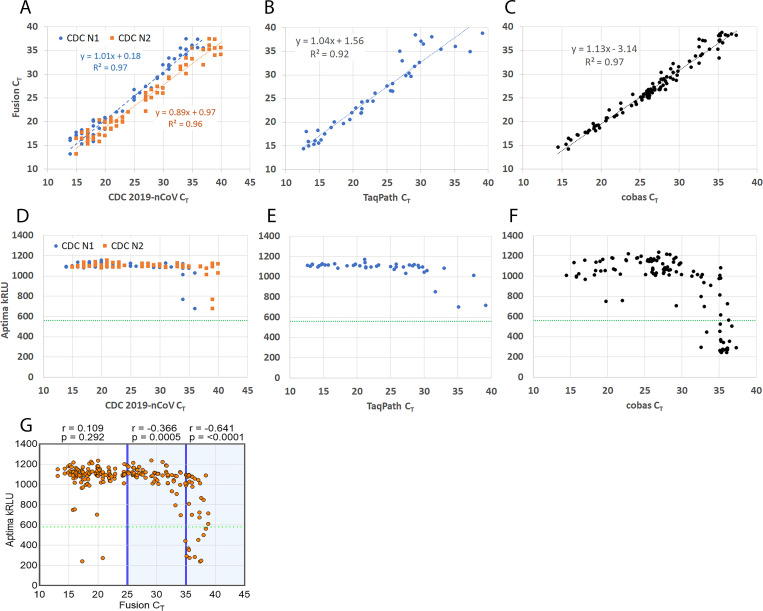
Fusion^®^ and Aptima^®^ vs CDC 2019-nCoV, TaqPath™ and cobas^®^ assay neat test results. C_T_ results for Fusion^®^ (*ORF1ab* target) vs (A) CDC 2019-nCoV (N1 and N2 targets), (B) TaqPath™ (*ORF1ab*) and (C) cobas^®^ (*ORF1ab*). Linear regression equation shown as y = R^2^ = Coefficient of regression. kRLU results for Aptima^®^ (*ORF1ab*) vs (D) CDC 2019-nCoV, (E) TaqPath™ and (F) cobas^®^. (G) Aptima^®^ vs Fusion^®^ SARS-CoV-2 results with correlation coefficient (r) calculated for high (C_T_ <25), moderate (25<C_T_<35) and low (C_T_ >35) viral load ranges. Statistical significance (p) calculated by two-sided t-test. (D-G) Aptima^®^ cut-off threshold (560 kRLU) shown in green.

The neat sample results for both Hologic assays were compared with the previous results from NIDDL and WRNMMC for neat sample testing. The Fusion^®^ C_T_ values were comparable to neat C_T_ values from the CDC 2019-nCoV (manual pooling, Fig 1A), TaqPath™ (manual pooling, Fig 1B) and cobas^®^ (automated pooling, Fig 1C) assays. The Aptima^®^ assay demonstrated 100% percent positive agreement (PPA) with CDC and TaqPath™ results, and 82% PPA with cobas^®^ results (Fig 1D–1F). There was 93% PPA between the Aptima^®^ and Fusion^®^ assays. The Aptima^®^ kRLU values have a nonlinear relationship with the quantitative Fusion^®^ results (Fig 1G).

#### Pooled testing PPA

**Fig 2:**
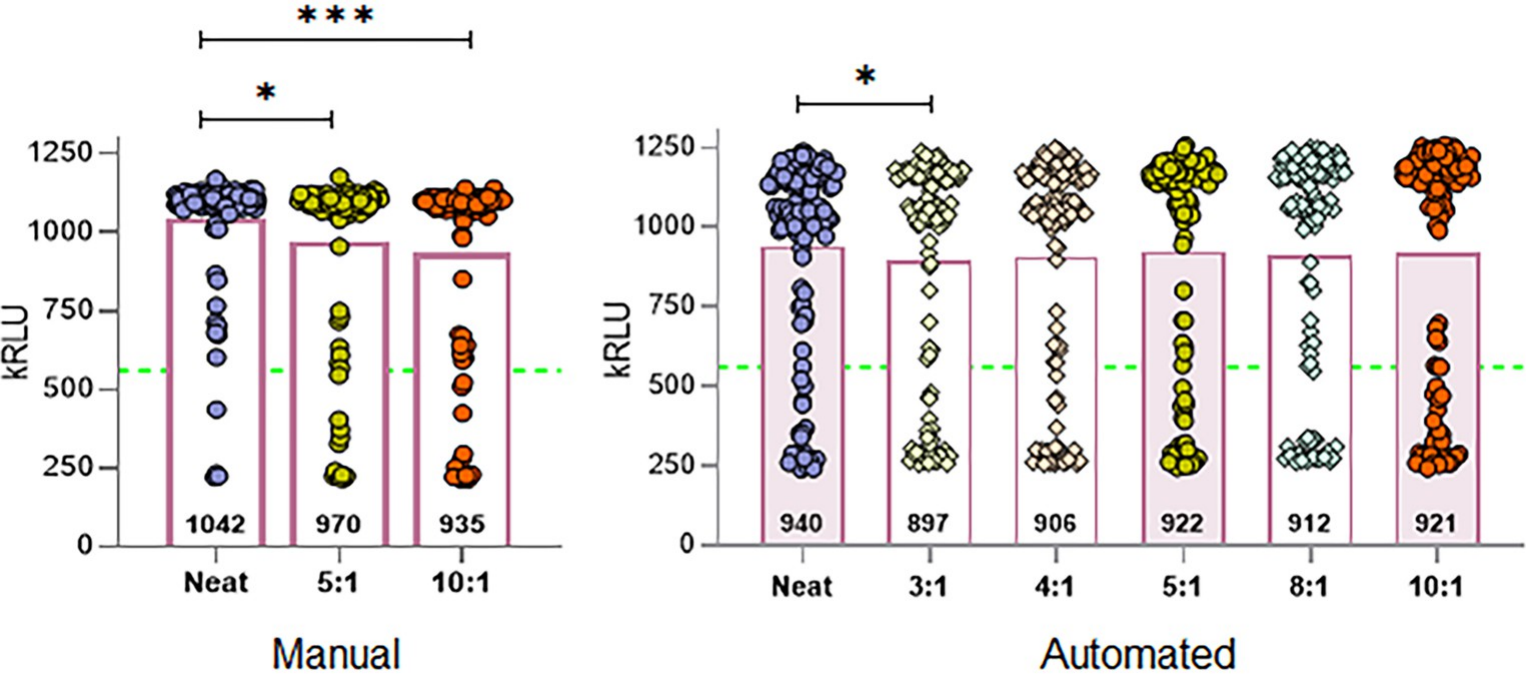
Distribution of Aptima^®^ pooled sample test results. Samples used in manual (left panel) and automated (right panel) pool generation. Test results are represented as circles. Numbers within bars represent median values. Dashed horizontal line represents cut-off value (560 kRLU). Two-sided t-test, * p<0.03, *** p<0.0001.

The Aptima^®^ assay demonstrated >85% within test PPA between neat and pooled sample test results for all pool sizes (Table 1, top row both Manual and Automated Pooling). However, when comparing Aptima^®^ automated pool sample results to results from the CDC, TaqPath™, and cobas^®^ assays, PPA values decreased by 9.9–10.4% (Table 1, right, middle row). Reducing the cut-off for Aptima^®^ from 560 kRLU to 350 kRLU led to improvement of overall PPA (>85%) for most pool sizes (Table 1, bottom row). Fig 2 shows the distribution of the Aptima^®^ SARS-CoV-2 pooled sample test results.

**Fig 3:**
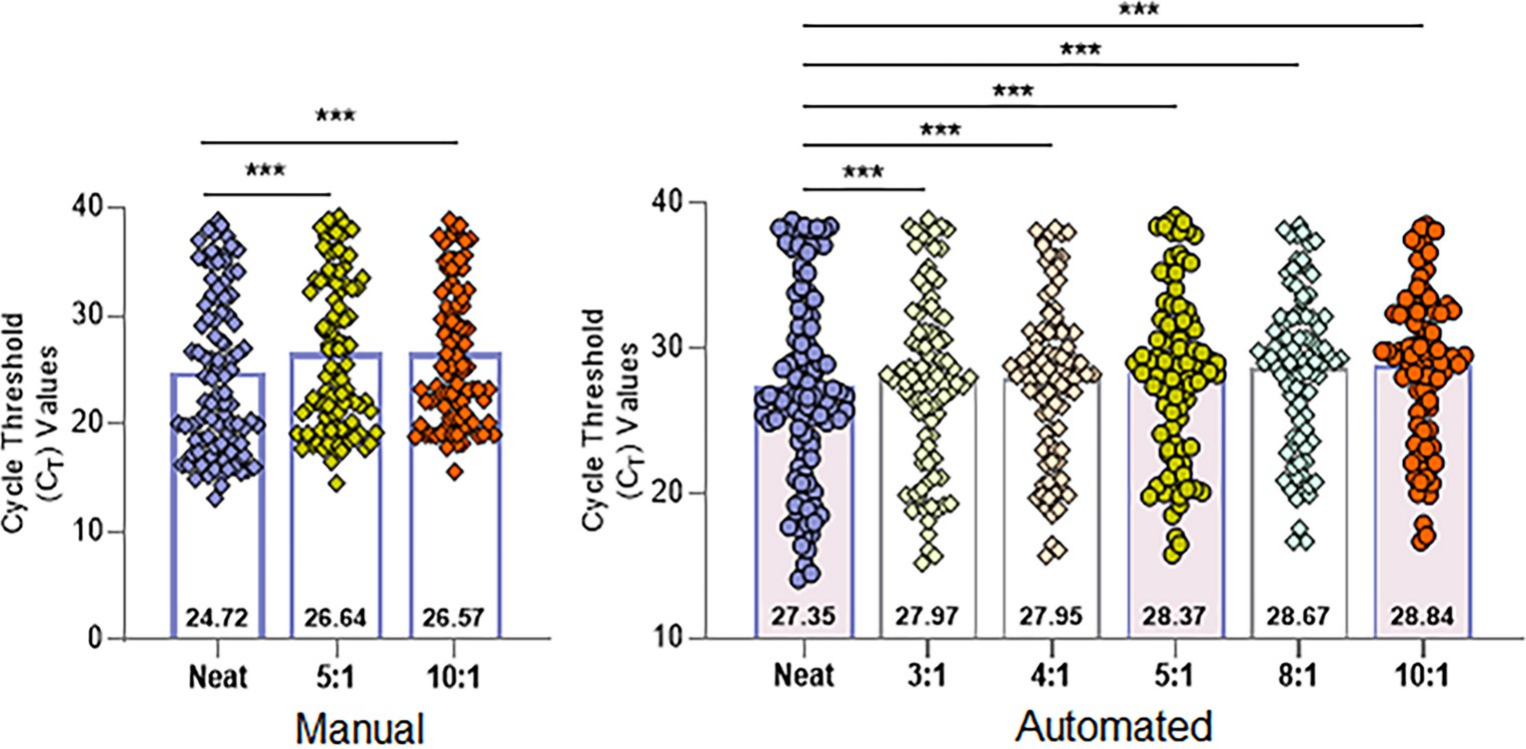
Distribution of Fusion^®^ neat and pooled sample test results. Samples used in manual pool generation (left panel) and automated pool generation (right panel). Test results are represented as circles. Numbers within bars represent median values. Two-sided t-test, ***p<0.0001.

The PPA between pooled and neat testing for Fusion^®^ was >85% for pool sizes ≤5:1, regardless of pooling method (Table 2, top row). For pool sizes >5:1, the PPA was <85% except for the manual pooling, 10:1 pool size scenario. The PPA results were similar when comparing the Fusion^®^ pooled test results to neat test results with the CDC, TaqPath™ and cobas^®^ assays (Table 2, bottom row). Fig 3 shows the distribution of the Fusion^®^ SARS-CoV-2 neat and pooled sample test results. The median C_T_ value increases as expected, and the distribution becomes more compressed for higher pool sizes.

Samples were categorized into three groups based on their Fusion^®^ C_T_ values: (1) C_T_ <25, high viral load; (2) 25 < C_T_ < 35, moderate viral load and (3) C_T_ > 35, low viral load (Fusion^®^ LLOD C_T_ = 35.6). Both assays performed well, regardless of pool size or pooling method. High and moderate viral load samples resulted in 94–100% PPA (Tables 3 and 4). Pooled test performance for samples with low viral loads was variable for both assays (Tables 3 and 4).

Test performance parameters for the Aptima^®^ and Fusion^®^ SARS-CoV-2 assays are presented with 95% confidence intervals in Tables 5 and 6. Both assays demonstrated high detection sensitivity, but the Aptima^®^ assay sensitivity was lower in the automated pooling study. Specificity of the Fusion^®^ SARS-CoV-2 assay was 4.3–10.1% lower than that of the Aptima^®^ assay except for the 4:1 pool size which yielded identical specificity by both assays. Cohen’s kappa coefficient (κ) values showed almost perfect agreement for the Aptima^®^ assay for pool sizes of 3:1, 4:1, 8:1 and 10:1 (Automated), with substantial agreement for all other pool sizes. The Fusion^®^ assay demonstrated near perfect agreement for only the 5:1 (manual) pool size.

#### C_T_ shift and linearity

**Fig 4:**
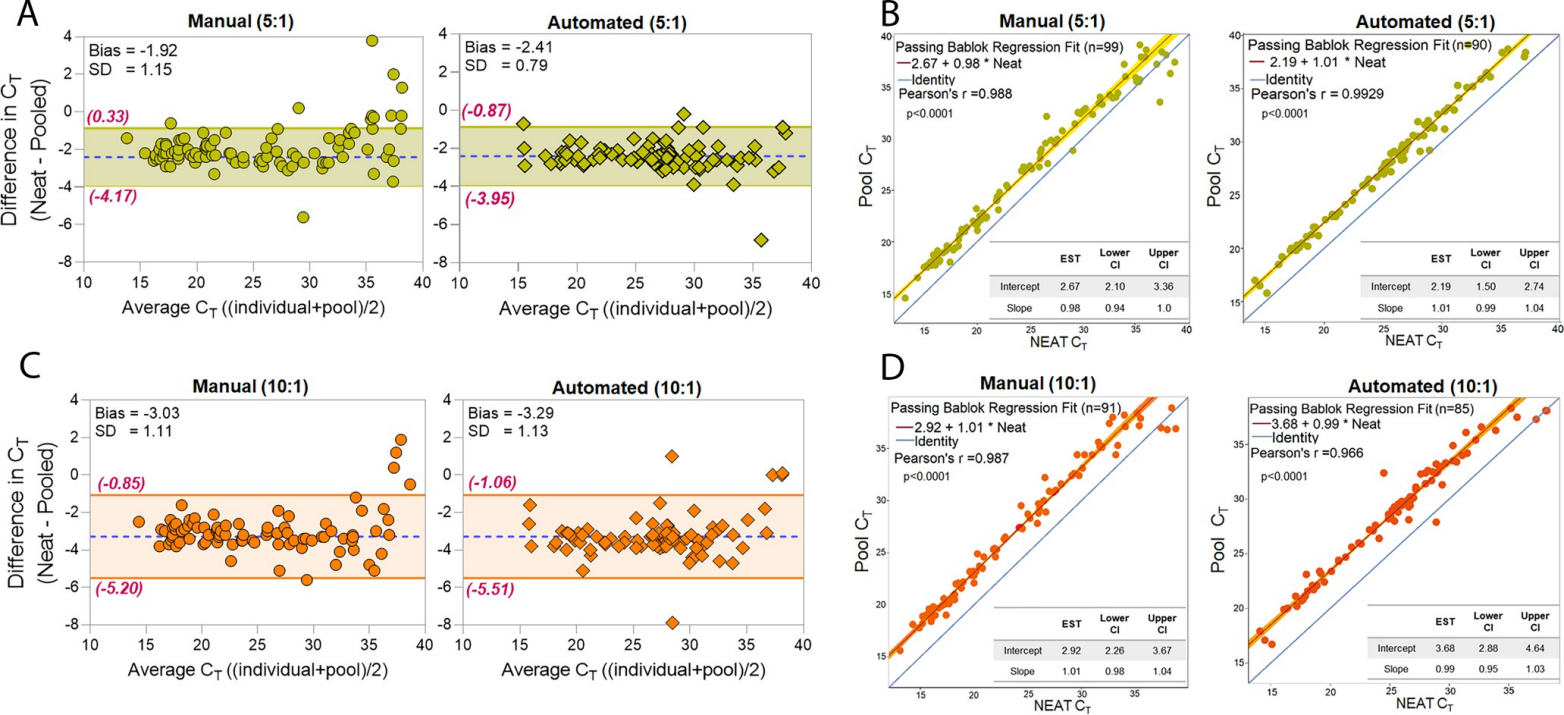
Linearity of the Panther Fusion^®^ SARS-CoV-2 assay for pooled testing. (A)(C) Bland-Altman plot and (B)(D) Passing-Bablok regression plots are presented for 5:1 or 10:1 pooled testing versus neat with the Panther Fusion^®^ SARS-CoV-2 assay. For the Bland-Altman plots, the area between the dotted lines (green or orange) indicates the 95% limits of agreement. For the Passing-Bablok regression, the confidence interval is shaded (green or orange) and the blue line indicates the line of identity. The slope and intercept of the regression line are reported in the left top of each panel.

A Bland-Altman plot showed good agreement for Fusion^®^ C_T_ results between the neat and pooled samples for both testing schemas (Fig 4A and 4C). For the 5:1 pool size, the C_T_ shift due to pooling is 1.91 (manual) and 2.41 (automated), while the 10:1 pool size had a C_T_ shift of 3.03 (manual) and 3.29 (automated).

Passing-Bablok regression fit indicates good linearity (slope confidence interval), with good agreement between the neat and pooled results for both 5:1 and 10:1 pool sizes (p<0.0001, Fig 4B and 4D).

### Source of RNA impacts LLOD

**Fig 5:**
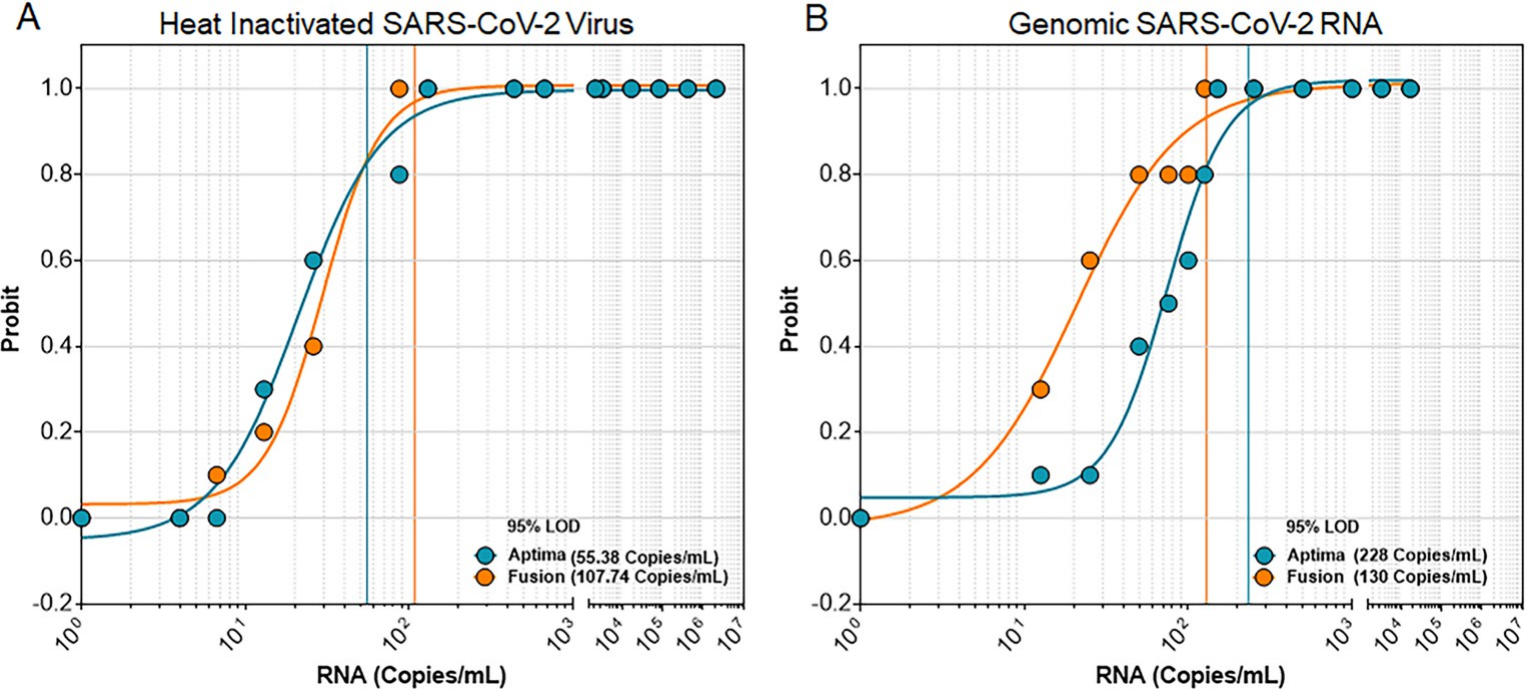
Source of RNA impacts LLOD. LLOD for the Hologic Panther^®^ SARS-CoV-2 assays determined by 5 independent measurements, performed over 5 days in duplicates (n = 10 for each datapoint). Blue: Aptima^®^ results. Orange: Fusion^®^ results. (A) Heat Inactivated Virus target in STM/UTM (B) Genomic RNA target in STM/UTM. The probit (predicted proportion of replicates positive) versus the SARS-CoV-2 RNA concentration identified.

In the heat inactivated virus + STM/UTM case, Aptima^®^ demonstrated a lower LLOD (55 copies/mL) than Fusion^®^ (108 copies/mL). However, when genomic RNA in STM/UTM was tested, Fusion^®^ yielded a lower LLOD (130 copies/mL) than Aptima^®^ (228 copies/mL) (Fig 5A and 5B).

### False positive (FP) results

The loss of detection sensitivity in pooled samples can be explained by dilution. However, several samples tested negative by neat testing, but positive when pooled. Four out of 32 samples that previously tested negative at a clinical lab, and yielded negative results by neat testing with both Hologic SARS-CoV-2 assays, tested positive when pooled (one 5:1 and three 10:1) using two-step pooled testing with the Fusion^®^ assay (S2A Fig). None of these four negative samples tested positive by pooled testing on the Aptima^®^ SARS-CoV-2 assay. Variable results were observed between one-step (direct) pooling and the two-step pooling processes (S2B Fig), with a single false positive result out of 25 negative samples tested. Automated pool generation showed lower incidence of false positive results for negative sample testing, with only a single false positive result by the Aptima^®^ assay at the 10:1 pool size (S2C Fig).

### Fusion^®^ assay less susceptible to interference between samples

To quantify interference from negative clinical discards, we calculated the 95% LLOD when using heat inactivated SARS-CoV-2 virus with either SARS-CoV-2 RNA negative post residual clinical test discards or STM/UTM to generate pools. Pools generated using post residual discards had a higher LLOD with the Aptima^®^ assay at all pool sizes except 10:1 (S3A Fig). An improvement in LLOD was also demonstrated if the Aptima^®^ cut-off was decreased from 560 kRLU to 350 kRLU (S3C Fig). Pooling with negative discards vs STM/UTM had mixed impact on the Fusion^®^ assay LLOD (S3B Fig). The increase in LLOD for pooled testing with the negative clinical samples indicates the presence of interference from negative clinical samples that is more problematic for the Aptima^®^ assay. While lowering the Aptima^®^ cut-off to 350 kRLU does not alleviate the interference caused by NP discards, lowering the Aptima^®^ cut-off does improve its LLOD to that of Fusion^®^ for pools sizes ≤5:1 (S3D Fig).

## Discussion

Pooling samples is a useful strategy to increase surveillance capability in order to identify SARS-CoV-2 asymptomatic individuals before they cause disease outbreaks. This study distinguishes itself from other pooled testing studies [7, 15–18] by combining the following features: large number of samples and pool sizes, comparing manual and automated methods of pooling samples, and analysis of pooled sample interaction for different nucleic acid amplification chemistries.

Our study compared both manual and automated methods of pooling samples. While many SARS-CoV-2 assays are run on high throughput automated systems that can process >1000 samples / day (Hologic Panther^®^, Roche cobas^®^), manual pooled sample generation can be rate-limiting [19]. Automated pooling of test samples improved test throughput and allowed testing of three additional pool sizes in our study (3:1, 4:1 and 8:1). Establishing automated pooling capability requires an initial investment in robotic liquid handlers and software development / verification. Software development and verification costs can be reduced by using open-source pooling scripts to implement sophisticated automated pooling systems for SARS-CoV-2 surveillance testing [20]. The average time to generate 16 pooled samples (5:1) was 6–7 minutes, including time required to load and unload samples. Up to 80 pooled samples (400 source samples) could be generated using the same strategy in approximately 24 minutes. A downstream test platform which can test 1000 pooled samples per day would require ~5 hours of automated sample pool generation.

Our pooling study tested retrospectively, while many other studies relied on prospective testing of pooled samples. With prospective testing, many of the positive pools may have more than one positive sample, thus resulting in positive pools with higher viral loads than retrospective testing. A study from Wang et al. [15] that characterized the effect of pooling on the analytical sensitivity of both the Hologic Fusion^®^ and Aptima^®^ SARS-CoV-2 assays generated only 36 (pool size 8) and 21 (pool size 4) positive pools with a single positive sample, limiting the statistical significance of their model. Another prospective pooled testing study from Migueres et al. [17] compared pooled sample testing of saliva samples with the Hologic Fusion^®^ and Aptima^®^ SARS-CoV-2 assays. This study has a similar weakness to the Wang et al. study, with only 18 positive pools with a single positive sample. However, they were able to demonstrate that Aptima^®^ assay sensitivity is comparable to Fusion^®^ for detecting SARS-CoV-2 in saliva samples.

Newsom et. al. [16] compared NP and saliva sample testing with the Aptima^®^ SARS-CoV-2 TMA assay and CDC 2019-NCoV2 RT-PCR assay for pooled testing of NP samples (pool size of 10), but not the Fusion^®^ assay. They showed that reducing the Aptima^®^ threshold from 560 kRLU to 324 kRLU was necessary to increase sensitivity and avoid false negative results for pooled testing. Our study focused on NP samples using both Aptima^®^ and Fusion^®^ assays, and our results also support Newsom’s findings that reducing the Aptima^®^ threshold increases sensitivity for pooled surveillance testing without impacting specificity. Barat et al. [7] examined testing pooled saliva samples using three different RT-PCR based assays, including the Panther Fusion^®^ SARS-CoV-2 assay, to increase participation in a voluntary screening program. They observed lower viral load in saliva vs NP samples, so they limited pool size to 5 instead of 10, which they had used with NP samples in a previous pooling study [18].

Our study demonstrated that the same test PPA between the individual and pooled sample results for the Aptima^®^ SARS-CoV-2 assay satisfied the FDA recommended guidelines of ≥85% [21] for pool sizes up to and including 10:1. However, these PPA results were associated with low sensitivity, especially in the automated pooling study. For the randomly sampled post residual clinical test specimens received from NIDDL and WRNMMC, 14–16% of the specimens had C_T_ >35. This proportion concurs with previous reports of >15% of first-time diagnostic specimens with C_T_>35 [15]. The automated pooling results had a higher proportion of moderate viral load samples (25<C_T_<35, 56% vs 35%) and lower proportion of high viral load samples (C_T_<25, 28% vs 51%) than the manual pooling results. In addition to evaluating the same test PPA as recommended by the FDA, we suggest validating the sensitivity of the assay used for pooling, demonstrated by Wunsch et al. [22] for PCR. The Fusion^®^ SARS-CoV-2 assay had a more consistent detection sensitivity across viral load distributions for both the manual and automated pooling. However, the Fusion^®^ assay’s PPA and sensitivity fell below 85% for pool sizes larger than 5:1 in the automated pooling scenarios. These results agree with the studies that recommend limiting pooled sample testing to pool sizes of 5:1 and align with the label’s intended use [10, 15, 23], and contrast with studies that promote testing with pool sizes larger than 5:1 [24, 25].

The FDA has granted an EUA for pooling up to 5 samples with the Hologic Aptima^®^ SARS-CoV-2 assay (FDA EUA200734). The Fusion^®^ SARS-CoV-2 assay demonstrated higher sensitivity and lower LLOD than the Aptima^®^ assay. However, lowering the cut-off threshold of the Aptima^®^ SARS-CoV-2 assay from 560 kRLU to 350 kRLU for pooled surveillance testing would improve its sensitivity and LLOD to that of Fusion^®^. Specimens with higher C_T_ >25 (moderate and low viremia) are most impacted by this shift in the cut-off value. Other studies that examined pooled surveillance testing with the Aptima^®^ SARS-CoV-2 assay recommended thresholds of 350 kRLU [15] or 324 kRLU [16]. However, lowering the cut-off value without taking kinetics into account could have other impacts to Aptima^®^ assay performance; therefore, further assessment would be required.

The expected C_T_ shift for a 5:1 dilution is 2.32; the Fusion^®^ SARS-CoV-2 assay showed a shift of 1.92 with manual pooling and 2.41 with automated pooling. The automated pooling shift was closer to the expected value, perhaps reflecting more accurate pipetting than with manual pooling. A similar result for the 10:1 dilution where the automated pooling C_T_ shift was closer to the expected value (3.30) was also observed.

Discrepancies between sensitivity were noted for the Hologic Panther^®^ assays, especially for the automated pooling study. The automated pooling study was conducted 6 months after the manual pooling study and used clinical discard specimens from WRNMMC instead of NIDDL. While some discrepancies are to be expected, the lower proportion of high viral load samples for the automated pool generation tests (28% vs 51% for the manual pool generation) reduced the calculated sensitivity of Aptima^®^ more than Fusion^®^. Since all specimens for both manual and automated pooling were collected in the same media type (VTM), albeit sourced from various vendors, the test performance was not likely affected by collection media.

This study may have been impacted by a few limitations. We were unable to confirm the false positive results with secondary testing due to insufficient specimen volumes. The specimens were freeze-thawed once, which could have impacted the results of one assay more than the other. The samples used for the manual pooling study were stored for 1–6 months longer than those used in the automated pooling study. We cannot make a direct comparison of C_T_ shifts due to different storage durations because the samples for manual pooling were initially tested with either the CDC RT-PCR for SARS-CoV-2 assay or the ThermoFisher TaqPath™ COVID-19 Combo assay, while the samples for automated pooling were initially tested with the Roche cobas^®^ SARS-CoV-2 assay.

Published LLODs from other studies range from 83–288 cp/mL [26, 27] for Aptima^®^ and from 62.5–125 cp/mL [28, 29] for Fusion^®^. Our analysis of LLOD for the Hologic SARS-CoV-2 assays (55–228 cp/mL for Aptima^®^ and 108–130 cp/mL for Fusion^®^, Fig 5) agree with published ranges.

Preliminary studies show that the FP rate for SARS-CoV-2 RT-PCR assays is between 0.8%-4.0% [30, 31]. Currently, there are no requirements nor recommendations for specificity analysis for pooled testing. We found FP test results were less common with automated pool generation and speculate it is due to decreased pipetting errors and reduced contamination risks. Further performance assessments are required to establish specificity of pooled testing for Hologic Panther^®^ assays.

While automated pooling offers many advantages, the high up-front and recurring costs required to establish and maintain an automated pooling system may be prohibitive for low resource settings.

## Conclusion

Surveillance testing of SARS-CoV-2 infections in U.S. military personnel requires high throughput testing platform capacity linked to high throughput pooling methods. We showed that TMA assays can be a viable alternative to RT-PCR assays for detection of SARS-CoV-2 in pooling schema. While pooled testing is a simple method to increase throughput for infection surveillance, there are trade-offs between throughput and detection sensitivity that depend not only upon infection prevalence, but platform chemistry. Linking automated sample process with high throughput test capability for large scale surveillance testing provides an approach which can be readily pivoted to survey for new emerging or reemerging pathogens.

## Supporting information

S1 Table Characteristics of specimens used for this study. (DOCX)

S2 Table Characteristics of the SARS-CoV-2 assays. (DOCX)

S3 Table Concentrations of heat inactivated SARS-CoV-2 virus used to generate concentrations to measure LLOD. (DOCX)

S1 Fig Pooled testing schema for testing with Hologic Panther Aptima^®^ and Fusion^®^ SARS-CoV-2 assays. (A) In the two-step process, the negative sample pools were generated first by mixing 4 or 9 samples in equal volume for the 5:1 and 10:1 pooled testing, respectively. From each pool, 400 μL (5:1) or 450 μL (10:1) was transferred into Hologic Panther^®^ SLT tubes, into which 100 μL (5:1) or 50 μL (10:1) of the uniquely identified positive sample was added to obtain the required testing volume of 500 μL for the Hologic Panther^®^ assays. (B) In the one-step process, the negative and positive samples were added directly into the SLT tubes in equal volume.
(PNG)

S2 Fig Negative sample testing. (A) Manual, two-step process used for pooled testing of 32 negative clinical discard specimens. False positive C_T_ results for Fusion^®^ shown (no FPs for Aptima^®^). (B) Comparison of direct pooling vs 2-step. (C) Automated generation of 200 pools (40 pools of each size) using negative specimens. The manufacturer’s Aptima^®^ cut-off (560 kRLU) and proposed cut-off (350 kRLU) shown.
(PNG)

S3 Fig Pooled testing with negative clinical discards is associated with higher interference than STM/UTM. 95% LLOD shown for testing with (A) Aptima^®^ (B) Fusion^®^ and (C) Aptima^®^ using a lower 350 kRLU threshold. (D) Comparison LLOD of Aptima^®^ vs Fusion^®^ for Negative Discards.
(PNG)
