# Supplementary figures and images for: Validation of SARS-CoV-2 pooled testing for surveillance using the Panther Fusion® system: Impact of pool size, automation, and assay chemistry
Source: PLoS One. 2022 Nov 7;17(11):e0276729. doi: 10.1371/journal.pone.0276729 (PMC9639840; doi:10.1371/journal.pone.0276729)

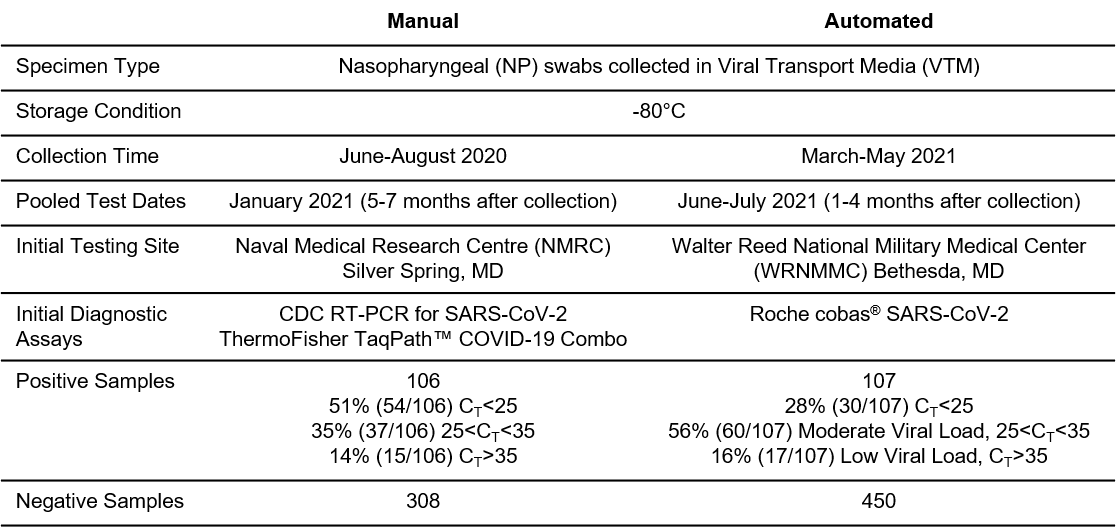


S1 Table. Characteristics of specimens used for this study

Supplement: S1 Table — (DOCX) [file pone.0276729.s001.docx]

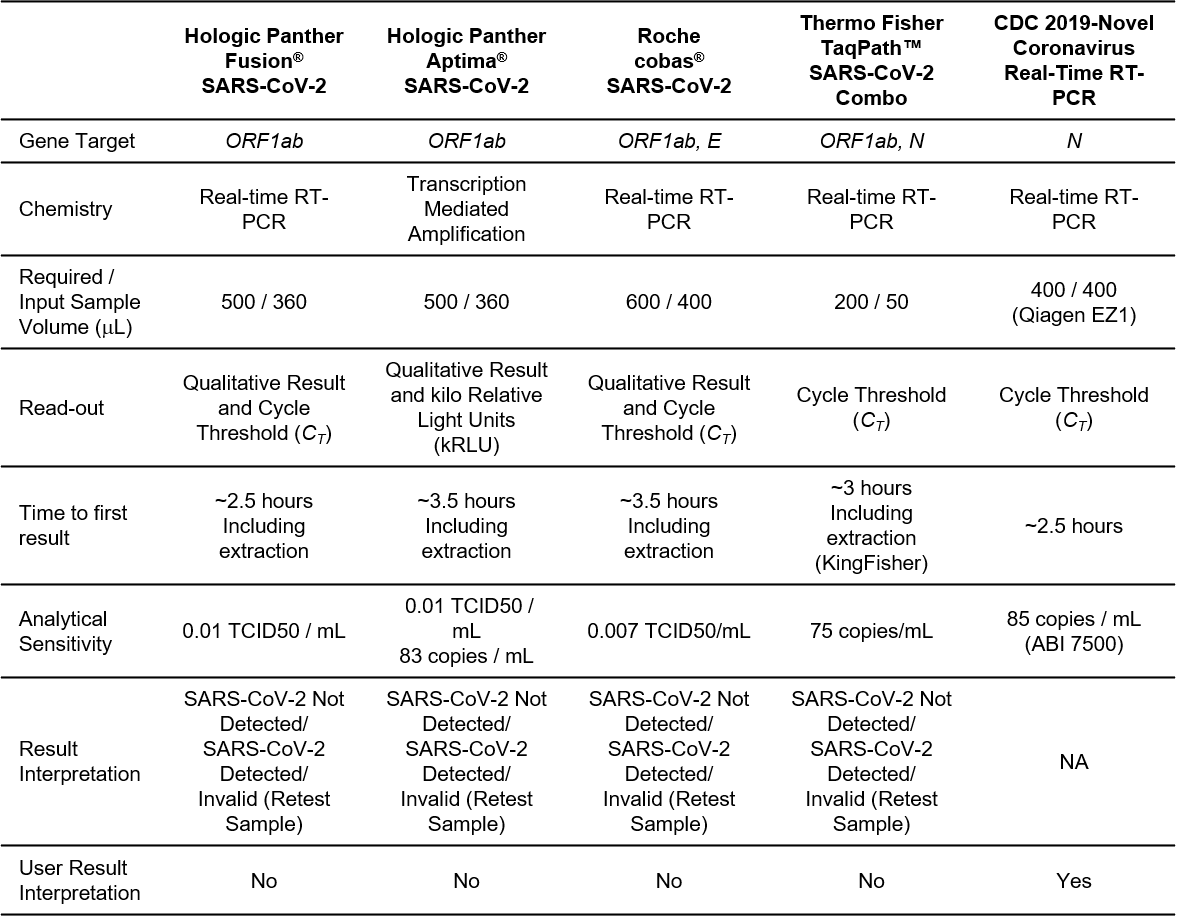


S2 Table. Characteristics of the SARS-CoV-2 assays.

Supplement: S2 Table — (DOCX) [file pone.0276729.s002.docx]

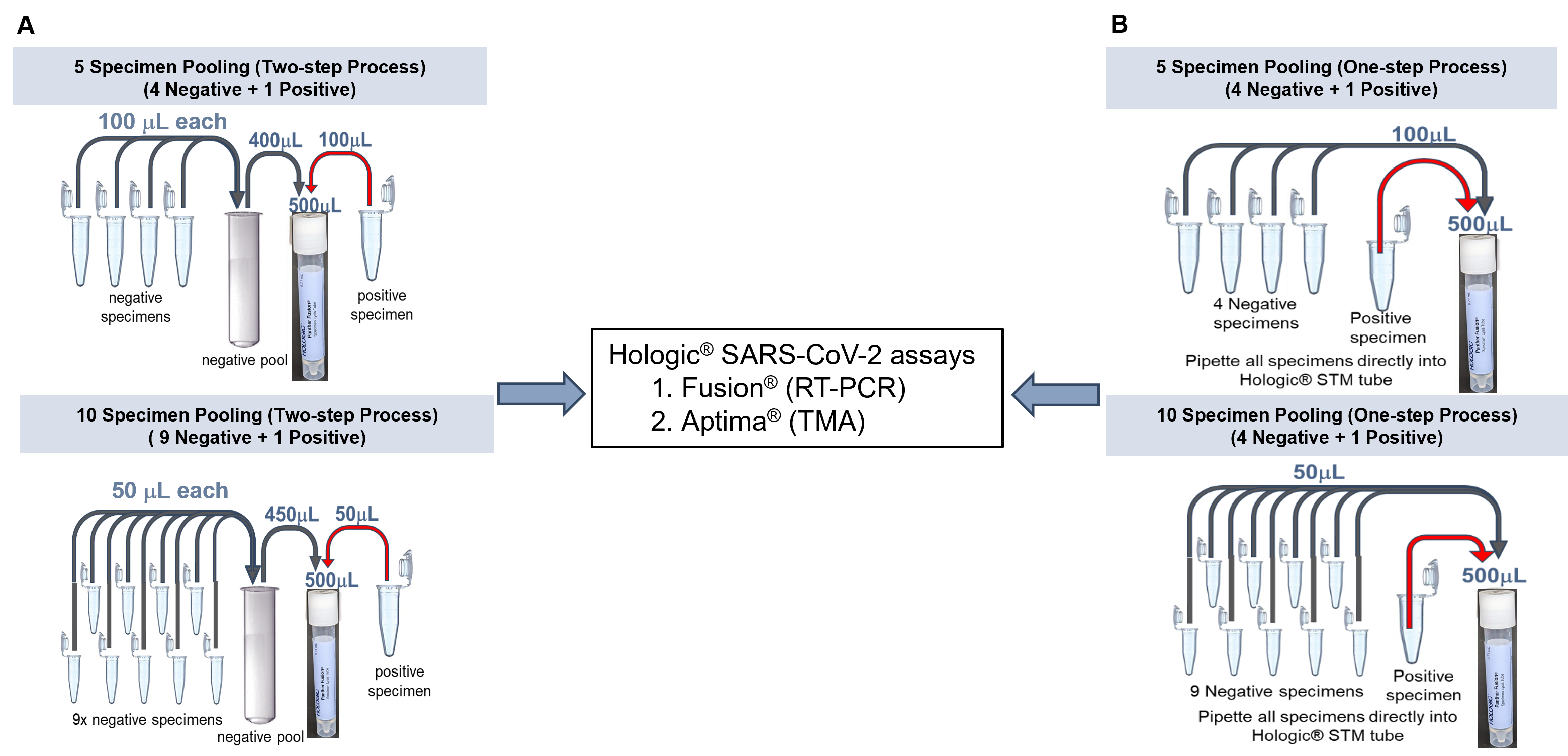

Supplement: S1 Fig — (A) In the two-step process, the negative sample pools were generated first by mixing 4 or 9 samples in equal volume for the 5:1 and 10:1 pooled testing, respectively. From each pool, 400 μL (5:1) or 450 μL (10:1) was transferred into Hologic Panther® SLT tubes, into which 100 μL (5:1) or 50 μL (10:1) of the uniquely identified positive sample was added to obtain the required testing volume of 500 μL for the Hologic Panther® assays. (B) In the one-step process, the negative and positive samples were added directly into the SLT tubes in equal volume. (PNG) [file pone.0276729.s004.png]

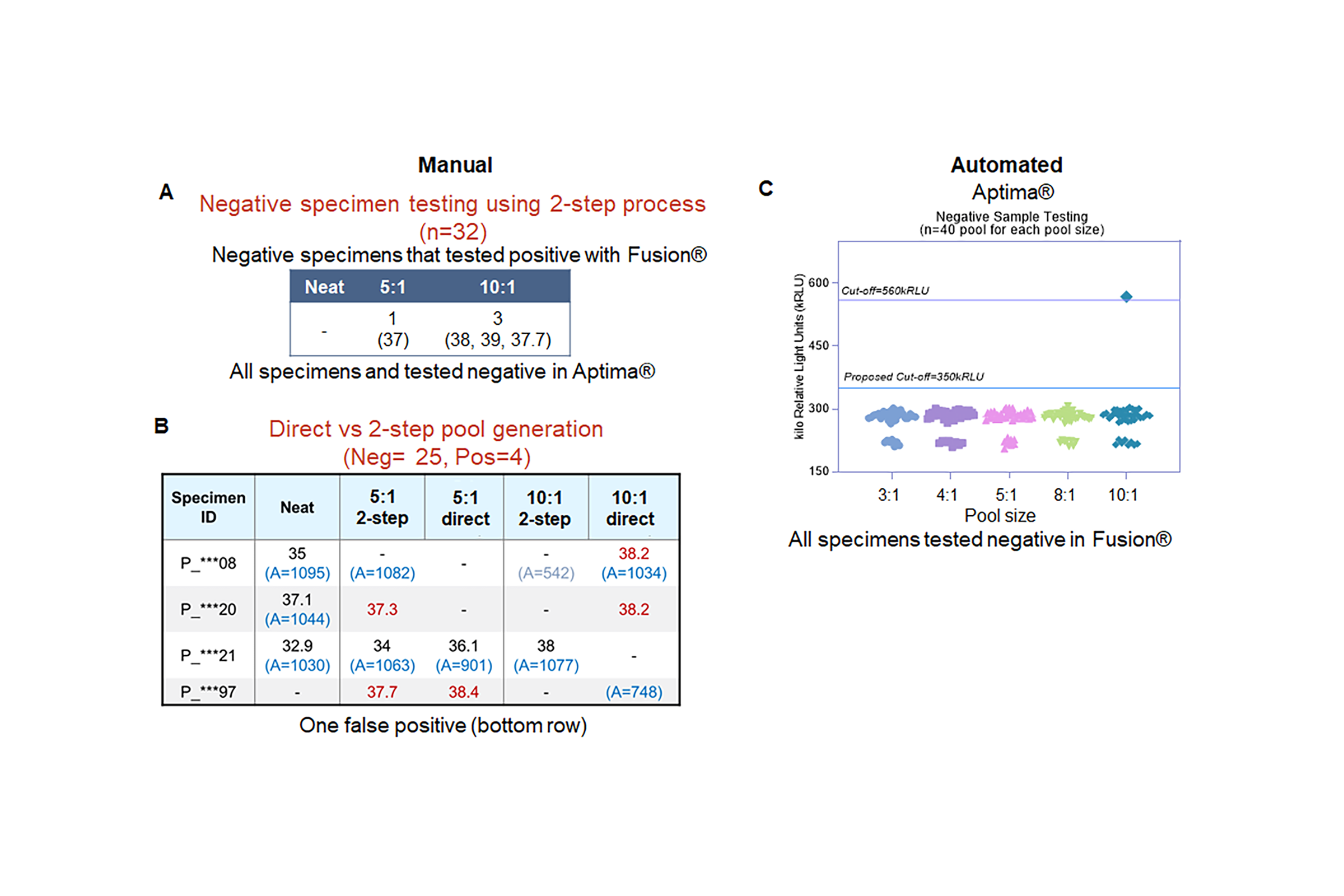

Supplement: S2 Fig — (A) Manual, two-step process used for pooled testing of 32 negative clinical discard specimens. False positive CT results for Fusion® shown (no FPs for Aptima®). (B) Comparison of direct pooling vs 2-step. (C) Automated generation of 200 pools (40 pools of each size) using negative specimens. The manufacturer’s Aptima® cut-off (560 kRLU) and proposed cut-off (350 kRLU) shown. (PNG) [file pone.0276729.s005.png]

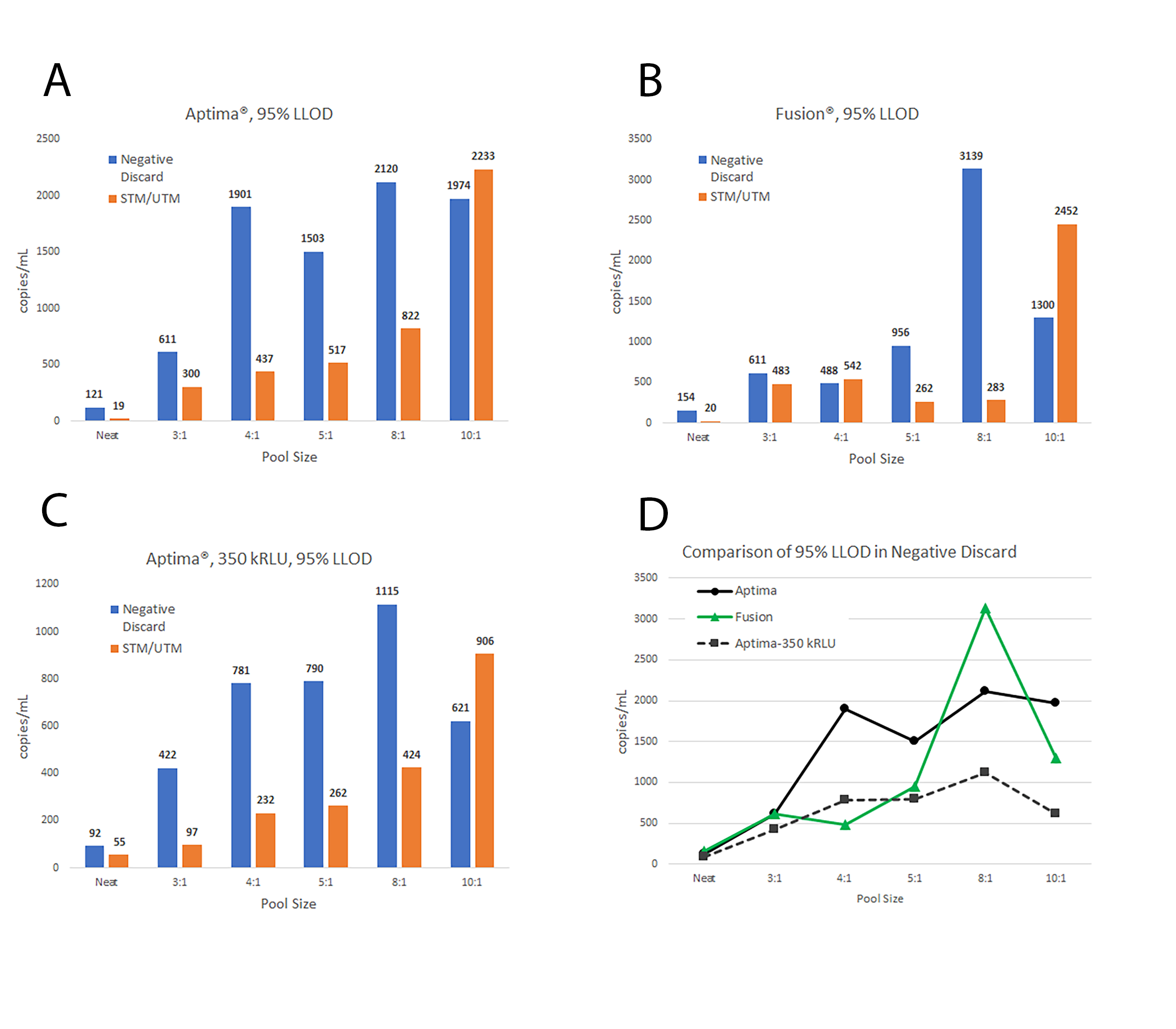

Supplement: S3 Fig — 95% LLOD shown for testing with (A) Aptima® (B) Fusion® and (C) Aptima® using a lower 350 kRLU threshold. (D) Comparison LLOD of Aptima® vs Fusion® for Negative Discards. (PNG) [file pone.0276729.s006.png]
