# Supplementary material for: Validation of SARS-CoV-2 pooled testing for surveillance using the Panther Fusion® system: Impact of pool size, automation, and assay chemistry
Source: PLoS One. 2022 Nov 7;17(11):e0276729. doi: 10.1371/journal.pone.0276729 (PMC9639840; doi:10.1371/journal.pone.0276729)
Supplement: S3 Table — (DOCX) [file pone.0276729.s003.docx]

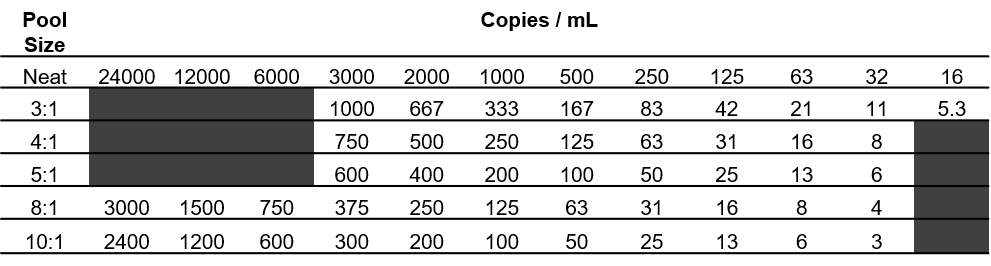


**S3 Table. Concentrations of heat inactivated SARS-CoV-2 virus used to generate concentrations to measure LLOD.**
